# Lipoprotein(a) is associated with the onset but not the progression of aortic valve calcification^[Author-notes ehac377-FM2]^

**DOI:** 10.1093/eurheartj/ehac377

**Published:** 2022-07-23

**Authors:** Yannick Kaiser, Janine E van der Toorn, Sunny S Singh, Kang H Zheng, Maryam Kavousi, Eric J G Sijbrands, Erik S G Stroes, Meike W Vernooij, Yolanda B de Rijke, S Matthijs Boekholdt, Daniel Bos

**Affiliations:** Department of Epidemiology, Erasmus MC, University Medical Center Rotterdam, Rotterdam, The Netherlands; Department of Vascular Medicine, Amsterdam Cardiovascular Sciences, Amsterdam UMC, University of Amsterdam, Amsterdam, The Netherlands; Department of Epidemiology, Erasmus MC, University Medical Center Rotterdam, Rotterdam, The Netherlands; Department of Radiology & Nuclear Medicine, Erasmus MC, University Medical Center Rotterdam, Rotterdam, The Netherlands; Department of Epidemiology, Erasmus MC, University Medical Center Rotterdam, Rotterdam, The Netherlands; Department of Internal Medicine, Erasmus MC, University Medical Center Rotterdam, Rotterdam, The Netherlands; Department of Clinical Chemistry, Erasmus MC, University Medical Center Rotterdam, Rotterdam, The Netherlands; Department of Vascular Medicine, Amsterdam Cardiovascular Sciences, Amsterdam UMC, University of Amsterdam, Amsterdam, The Netherlands; Department of Epidemiology, Erasmus MC, University Medical Center Rotterdam, Rotterdam, The Netherlands; Department of Internal Medicine, Erasmus MC, University Medical Center Rotterdam, Rotterdam, The Netherlands; Department of Vascular Medicine, Amsterdam Cardiovascular Sciences, Amsterdam UMC, University of Amsterdam, Amsterdam, The Netherlands; Department of Radiology & Nuclear Medicine, Erasmus MC, University Medical Center Rotterdam, Rotterdam, The Netherlands; Department of Clinical Chemistry, Erasmus MC, University Medical Center Rotterdam, Rotterdam, The Netherlands; Department of Cardiology, Amsterdam Cardiovascular Sciences, Amsterdam UMC, University of Amsterdam, Amsterdam, The Netherlands; Department of Epidemiology, Erasmus MC, University Medical Center Rotterdam, Rotterdam, The Netherlands; Department of Radiology & Nuclear Medicine, Erasmus MC, University Medical Center Rotterdam, Rotterdam, The Netherlands

**Keywords:** Lipoprotein(a), Aortic valve calcium, Aortic valve stenosis, Cardiac CT

## Abstract

**Aim:**

Lipoprotein(a) [Lp(a)] is a potential causal factor in the pathogenesis of aortic valve disease. However, the relationship of Lp(a) with new onset and progression of aortic valve calcium (AVC) has not been studied. The purpose of the study was to assess whether high serum levels of Lp(a) are associated with AVC incidence and progression.

**Methods and results:**

A total of 922 individuals from the population-based Rotterdam Study (mean age 66.0±4.2 years, 47.7% men), whose Lp(a) measurements were available, underwent non-enhanced cardiac computed tomography imaging at baseline and after a median follow-up of 14.0 [interquartile range (IQR) 13.9–14.2] years. New-onset AVC was defined as an AVC score >0 on the follow-up scan in the absence of AVC on the first scan. Progression was defined as the absolute difference in AVC score between the baseline and follow-up scan. Logistic and linear regression analyses were performed to evaluate the relationship of Lp(a) with baseline, new onset, and progression of AVC. All analyses were corrected for age, sex, body mass index, smoking, hypertension, dyslipidaemia, and creatinine. AVC progression was analysed conditional on baseline AVC score expressed as restricted cubic splines. Of the 702 individuals without AVC at baseline, 415 (59.1%) developed new-onset AVC on the follow-up scan. In those with baseline AVC, median annual progression was 13.5 (IQR = 5.2–37.8) Agatston units (AU). Lipoprotein(a) concentration was independently associated with baseline AVC [odds ratio (OR) 1.43 for each 50 mg/dL higher Lp(a); 95% confidence interval (CI) 1.15–1.79] and new-onset AVC (OR 1.30 for each 50 mg/dL higher Lp(a); 95% CI 1.02–1.65), but not with AVC progression (*β*: −71 AU for each 50 mg/dL higher Lp(a); 95% CI −117; 35). Only baseline AVC score was significantly associated with AVC progression (*P* < 0.001).

**Conclusion:**

In the population-based Rotterdam Study, Lp(a) is robustly associated with baseline and new-onset AVC but not with AVC progression, suggesting that Lp(a)-lowering interventions may be most effective in pre-calcific stages of aortic valve disease.

## Structured Graphical Abstract

**Structured Graphical Abstract:**
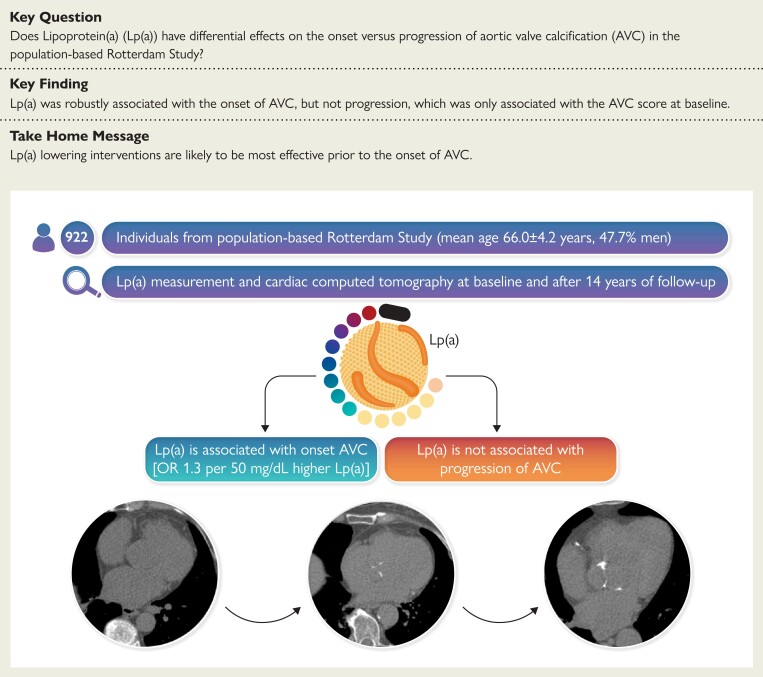

In the population-based Rotterdam Study, lipoprotein(a) [Lp(a)] was associated with aortic valve calcification (AVC) onset in multivariable analysis adjusted for age, sex, body mass index, smoking, dyslipidaemia, hypertension, and creatinine. In contrast, progression of AVC was only associated with baseline AVC score, implying that disease progression may take place independently of initiating risk factors. AVC, aortic valve calcification; Lp(a), lipoprotein(a).

**See the editorial comment for this article ‘Lipoprotein(a) and aortic valve stenosis: work in progress’, by Florian Kronenberg, https://doi.org/10.1093/eurheartj/ehac436.**

## Introduction

Aortic valve stenosis (AVS) is an increasingly prevalent condition in our aging society.^1^ Its presence can remain asymptomatic for decades until significant valvular stenosis leads to clinical sequelae such as syncope, heart failure, and even sudden death.^2^ One in seven AVS cases can be attributed to elevated lipoprotein(a) [Lp(a)] levels,^3^ and genetic studies suggest that Lp(a) is a causal factor in the aetiology of AVS.^4–6^ Lipoprotein(a)-lowering strategies have consequently been put forward as a promising strategy to attenuate AVS progression. Although selective Lp(a)-lowering therapies^7^ are currently under evaluation in a Phase 3 cardiovascular prevention trial (NCT04023552), there are no trials in AVS yet, as it remains unclear whether Lp(a) is associated with progression of established valvular calcifications.

The presence of aortic valve calcification (AVC) detected by computed tomography (CT) is the earliest discernable stage of aortic valve disease, even before functional stenosis begins to develop.^8^ Moreover, serial CT AVC quantification has been validated as a reproducible method to assess AVC, and performed better than echocardiographic measures to detect disease progression.^9^ In order to assess the feasibility of Lp(a)-lowering trials in AVS, data are required on the relationship between Lp(a) and AVC progression, because established calcium content within the aortic valve is a powerful driver of further disease progression.^10,11^ For instance, a previous analysis in the Multi-Ethnic Study of Atherosclerosis (MESA) demonstrated that although traditional risk factors such as low-density lipoprotein-cholesterol (LDL-C) are strongly associated with the onset of AVC, they were not associated with AVC progression, which was only determined by baseline AVC score.^12^ Consistently, LDL-C-lowering interventions in AVS have been unable to attenuate disease progression in patients with established calcifications.^13–15^

We hypothesized that Lp(a) is associated with the onset AVC but less so with progression in individuals with established AVC. Therefore, we measured serum Lp(a) and performed serial non-enhanced cardiac CT in the population-based Rotterdam Study.

## Methods

### Study participants

**Figure 1:**
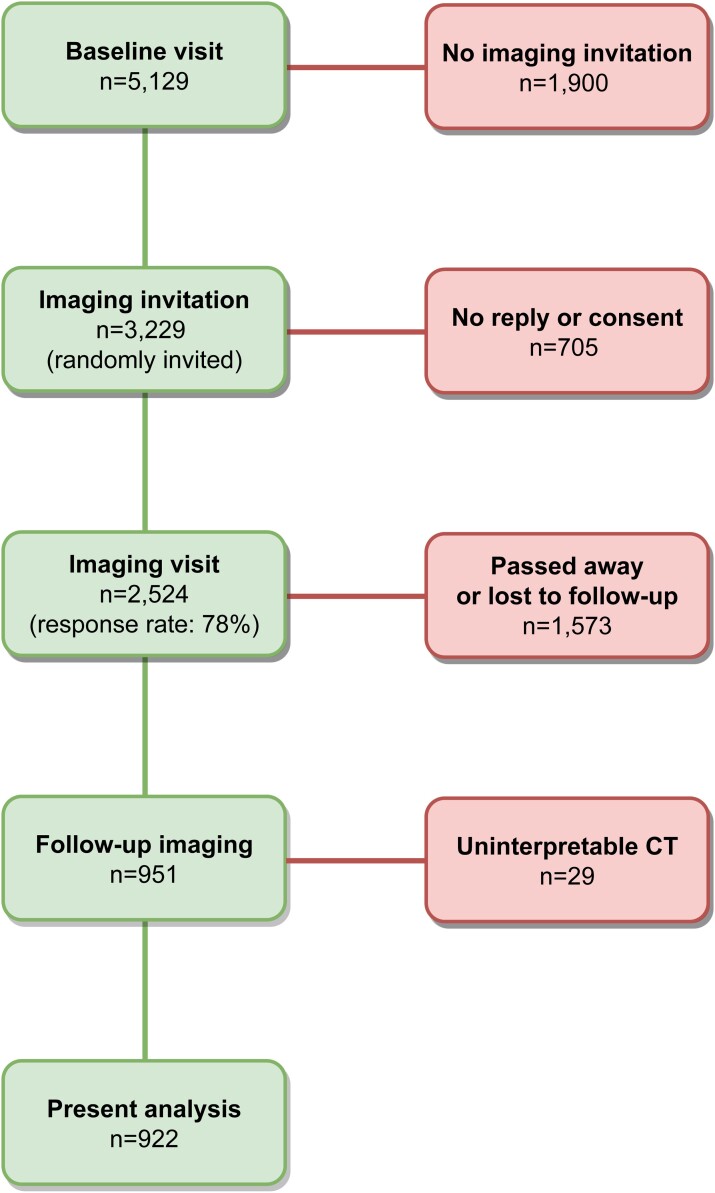
Study flow chart.

The Rotterdam Study is an ongoing population-based cohort study aimed to investigate determinants of age-related disease and currently includes >15 000 individuals aged ≥40 years. The study design and recruitment process have previously been reported in detail.^16^ In the present study, we took the visit between 2003 and 2006 as baseline (*n* = 5129), from whom a random sample of 3229 individuals was invited to undergo non-contrast enhanced multidetector CT (MDCT). A total of 2524 individuals participated in this first imaging visit. Between 2018 and 2019, all individuals who underwent prior imaging were invited for follow-up imaging, out of whom 951 underwent the follow-up scan. A flow chart of the study is shown in *Figure 1*. The Rotterdam Study has been approved by the Medical Ethics Committee of the Erasmus MC. The Rotterdam Study has been entered into The Netherlands National Trial Register (NTR; www.trialregister.nl) and into the WHO International Clinical Trials Registry Platform under shared catalogue number NTR6831. All participants provided written informed consent to participate in the study.

### Image acquisition and analysis

At the baseline examination, non-contrast CT images were obtained using 16- or 64-slice MDCT scanners (Somatom Sensation 16 or 64; Siemens, Forchheim, Germany). To quantify AVC, a cardiac scan was performed that ranged from the apex of the heart to the tracheal bifurcation. Cardiac images were obtained within a single breath hold. For the 16-slice scanner, the scan parameters were: 12 × 1.5 mm collimation, 120 kVp, effective 30 mAs, and prospective electrocardiogram triggering at 50% of the cardiac cycle. For the 64-slice scanner, parameters were similar except that the collimation was 32 × 0.6 mm, and the mAs value was real-time adapted to body weight (CARE Dose 4D, Siemens). Further information on the scan protocol is provided elsewhere.^16^

At the second examination, non-contrast CT images were obtained using a 128-slice dual-source CT (DSCT) scanner (Somatom Drive; Siemens). Cardiac images were acquired within a single breath hold with 64 × 0.6 mm collimation, 120 kVp, and effective 80 mAs (CARE Dose 4D).

Aortic valve calcification was quantified using Agatston methodology^17^ by trained readers who were blinded to clinical data. The presence of AVC was defined as an AVC score >0 Agatston units (AU). Aortic valve calcification progression was defined as the absolute difference between the AVC score of the follow-up minus the baseline scan.

### Measurement of covariables

At the time of the baseline CT examination, relevant cardiovascular risk factors were assessed by interview (smoking, medication), physical examination (body mass index, blood pressure), or blood sampling (lipid panel and creatinine), according to previously described methodology.^16^ Dyslipidaemia was defined as non-high-density lipoprotein-cholesterol (non-HDL-C) >5 mmol/L and/or use of lipid-lowering medication. Hypertension was defined as a systolic blood pressure >140 mmHg, and/or diastolic blood pressure >90 mmHg, and/or use of blood pressure–lowering medication.

### Measurement of lipoprotein(a)

Lipoprotein(a) was measured in 2019 in plasma isolated from fasting baseline blood samples, which were stored at −80°C. Lipoprotein(a) measurement was performed on a Cobas 8000 analyser using an assay with minimal isoform-dependent bias due to the use of calibrators with differently sized apo(a) isoforms (Randox immunoassay; Randox Laboratories Ltd., UK).

### Statistical analysis

Baseline characteristics are reported as mean ± standard deviation (SD), median (interquartile range) or number (percentage), as appropriate. Three groups were defined based on the presence of AVC on baseline and follow-up: (i) no AVC on either scan, (ii) AVC onset defined as AVC only on the follow-up scan, and (iii) AVC on both scans. Between-group comparisons were tested with a one way independent analysis of variance test for parametric data, Kruskal–Wallis test for non-parametric data, and χ^2^ test for categorical data. The association of Lp(a) with baseline and development of AVC was assessed using multivariable logistic regression analysis, adjusting for known AVC risk factors at baseline: age, sex, body mass index, smoking, dyslipidaemia, hypertension, and creatinine. The association of Lp(a) with AVC progression was assessed using restricted cubic spline regression analysis, using the same models as mentioned previously, with additional adjustment for baseline AVC score expressed using restricted cubic splines with 2 knots, placed at 100 and 500 AU, to assess non-linearity. Participants who underwent valvular replacement were excluded from the primary AVC progression analysis. However, we performed a sensitivity analysis including individuals who underwent aortic valve replacement (AVR) by imputing the highest AVC score of the follow-up scan from our cohort. To aid the interpretation of the regression analyses, effect sizes were determined for each 50 mg/dL higher Lp(a), 5-year higher age, and an SD higher in normally distributed variables. Missing data percentages were 2.4% for smoking status, 15.0% for creatinine levels, 0.8% for hypertension, and 1.3% for lipid-lowering medication. There were no missing data for age, sex, Lp(a) levels, AVC, body mass index, and non-HDL-C. We used Little’s Missing Completely at Random test to validate whether data were missing at random. Data missing at random were imputed using the MICE package. Statistical testing was two sided with significance set at *α* = 0.05. All statistical analyses were performed using R version 4.0.5 (R Foundation, Vienna, Austria).

## Results

### Baseline characteristics

From the 951 participants with two CT examinations, 29 participants had an uninterpretable CT at either baseline or follow-up, leaving a total of 922 individuals included in the current analysis. Mean age was 66.0 ± 4.2 years and 440 (47.7%) individuals were men. Baseline characteristics are listed in *Table 1*, stratified by the presence of AVC on baseline and follow-up. Aortic valve calcification was present at baseline in 220 (23.9%) individuals with a median AVC score of 52 AU (15–131). These individuals were older, more often men, had higher Lp(a) and creatinine levels, lower HDL-C, and used lipid-lowering medication more frequently than participants who did not have AVC at baseline. Supplementary material online, *Table S1* shows baseline characteristics of the entire study cohort compared with those included in the present analysis.

**Table 1:** Baseline characteristics

	No AVC at baseline and follow-up (*n* = 287)	No AVC at baseline, AVC at follow-up (*n* = 415)	AVC at baseline and follow-up (*n* = 220)	*P*-value
Age (years)	64.5 ± 3.4	66.1 ± 4.3	67.7 ± 4.4	<0.001
Male	108 (37.6)	197 (47.5)	135 (61.4)	<0.001
Active smoker	28 (9.9)	58 (14.5)	28 (13.0)	0.200
Body mass index (kg/m^2^)	27.0 ± 3.5	27.8 ± 3.8	28.2 ± 3.6	0.001
Systolic blood pressure (mmHg)	141 ± 17	143 ± 18	144 ± 18	0.093
Diastolic blood pressure (mmHg)	81 ± 9	81 ± 10	81 ± 10	0.955
Total cholesterol (mmol/L)	5.74 ± 0.89	5.83 ± 0.96	5.70 ± 1.00	0.224
High-density lipoprotein-cholesterol (mmol/L)	1.51 ± 0.41	1.46 ± 0.38	1.36 ± 0.37	<0.001
Non-high-density lipoprotein-cholesterol (mmol/L)	4.23 ± 0.87	4.36 ± 0.95	4.34 ± 1.00	0.171
Lipoprotein(a) (mg/dL)	10 (5, 28)	13 (5, 38)	13 (6, 59)	0.024
Creatinine (mmol/L)	78 ± 15	80 ± 15	82 ± 16	0.010
Use of blood pressure–lowering medication	47 (16.5)	83 (20.3)	64 (29.5)	0.002
Use of lipid-lowering medication	82 (28.9)	134 (32.8)	78 (35.9)	0.237
Aortic valve calcium score on baseline (Agatston units)	0 (0, 0)	0 (0, 0)	52 (15, 131)	<0.001
Aortic valve calcium score on follow-up (Agatston units)	0 (0, 0)	33 (9, 103)	241 (118, 667)	<0.001

Values are original, non-imputed data, depicted as mean ± standard deviation for normally distributed data, median (interquartile range) for non-normally distributed data, and as number (percentage) for categorical data.

### Follow-up scan

**Figure 2:**
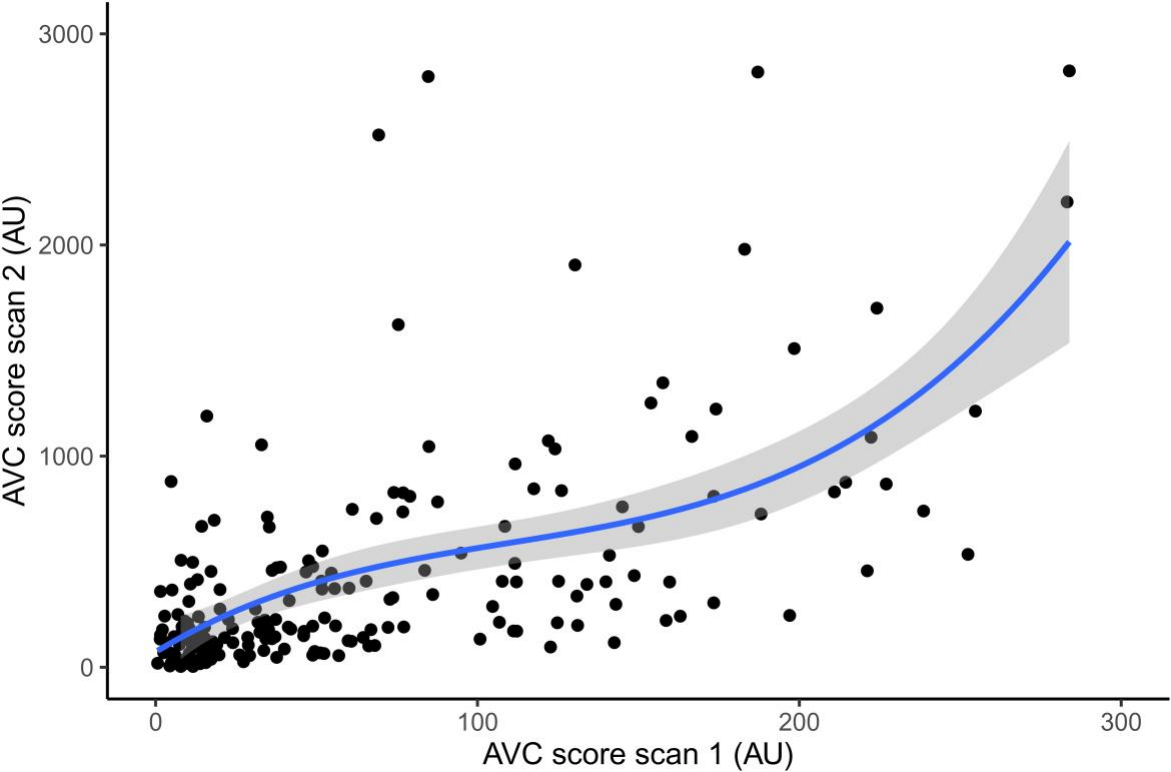
Progression rate of aortic valve calcification. Depicted is the relationship between the aortic valve calcification score at baseline and the aortic valve calcification score after 14 years of follow-up. AVC, aortic valve calcification; AU, Agatston units.

The median time from baseline to follow-up scan was 14.0 (13.9–14.2) years. Of the 702 participants without AVC on the baseline scan, 415 (59.1%) developed AVC on the follow-up scan, with a median AVC score of 32.7 AU (9.0–102.8). Similar to individuals with baseline AVC, these participants were older, more often men, had a higher body mass index, higher Lp(a) and creatinine levels, lower HDL-C, and used lipid-lowering medication more often than individuals who did not develop AVC. Among the 220 individuals with AVC at baseline, we observed a median annual progression of 13.5 AU (5.2–37.8), resulting in a median AVC score of 242.3 AU (121.7–667.5) on the follow-up scan. There were no individuals with AVC regression. Progression rates stratified by baseline AVC score are depicted in *Figure 2*.

### Relationship of lipoprotein(a) with baseline, new-onset, and progression of aortic valve calcification

The results of the multivariable adjusted regression analyses evaluating the relationship of Lp(a) with baseline, new onset and progression of AVC are listed in *Table 2*.

**Table 2:** Relationship of lipoprotein(a) with baseline, onset, and progression of aortic valve calcification

	Baseline aortic valve calcification OR (95% CI)	*P*-value	Onset of aortic valve calcification OR (95% CI)	*P*-value	Progression of aortic valve calcification^a^ Beta (95% CI)	*P*-value
Lipoprotein(a) (per 50 mg/dL higher)	1.43 (1.15–1.79)	0.001	1.30 (1.02–1.65)	0.036	−71 (−177; 35)	0.186
Age (per 5 years higher)	1.88 (1.56–2.26)	<0.001	1.86 (1.48–2.33)	<0.001	38 (−55; 131)	0.425
Male sex	2.07 (1.47–3.36)	<0.001	1.66 (1.11–2.48)	0.014	−56 (−257; 146)	0.586
Body mass index (per SD higher)	1.26 (1.07–1.48)	0.006	1.27 (1.08–1.50)	0.004	1 (−81; 84)	0.976
Active smoking	1.14 (0.70–1.84)	0.599	1.77 (1.08–2.90)	0.024	17 (−224; 257)	0.892
Dyslipidaemia	1.46 (1.05–2.03)	0.023	1.26 (0.91**–**1.75)	0.170	−79 (−253; 95)	0.373
Hypertension	1.06 (0.70**–**1.84)	0.756	1.00 (0.72**–**1.41)	0.969	−165 (−347; 17)	0.075
Creatinine (per SD higher)	1.05 (0.86**–**1.29)	0.619	0.95 (0.78**–**1.16)	0.609	67 (−31; 166)	0.179

Data are depicted as adjusted odds ratios (ORs) with 95% confidence intervals (CI) for baseline and onset of aortic valve calcification, and betas with 95% CI for aortic valve calcium score progression, measured in Agatston units (AU).

a The progression analysis is also adjusted for baseline aortic valve calcium score using restricted cubic splines with 2 knots, placed at 100 and 500 AU, respectively, which was the only variable associated with progression of aortic valve calcification (*P* < 0.001). *R*^2^ of the progression model = 0.58.

Lipoprotein(a) was significantly associated with baseline AVC [odds ratio (OR) 1.43/50 mg/dL higher Lp(a), 95% confidence interval (CI) 1.15–1.79] in multivariable logistic regression analysis. Other known risk factors for AVC associated with baseline AVC in the multivariable analysis included age (OR 1.88/5 years higher age, 95% CI 1.56–2.26), male sex (OR 2.07, 95% CI 1.47–3.36), body mass index (OR 1.26/SD higher, 95% CI 1.07–1.48), and dyslipidaemia (OR 1.46, 95% CI 1.05–2.03).

Lipoprotein(a) was also significantly associated with AVC onset on follow-up CT, with an effect size comparable with that for baseline AVC (OR 1.30/50 mg/dL higher Lp(a), 95% CI 1.02–1.65). Other risk factors associated with AVC onset were also similar to those for baseline AVC and included age (OR 1.86/5 years higher age, 95% CI 1.48–2.33), male sex (OR 1.66, 95% CI 1.11–2.48), body mass index (OR 1.27/SD higher, 95% CI 1.08–1.50), and active smoking (OR 1.77, 95% CI 1.08–2.90).

In contrast to baseline and new-onset AVC, Lp(a) was not associated with AVC progression (β-71 AU per 50 mg/dL higher Lp(a); 95% CI: −177; 35), nor were any traditional cardiovascular risk factors. In fact, baseline AVC score was the only parameter significantly associated with accelerated AVC progression (*P* < 0.001).

### Sensitivity analyses

A total of 14 individuals underwent AVR during follow-up, all of whom had AVC on the baseline scan. Their median baseline AVC score was 1175 AU (466–2448), the lowest baseline AVC score being 80 AU. Imputation of the highest AVC score on the follow-up scan in individuals who underwent AVR did not meaningfully change the results of the progression analysis (data not shown).

We also assessed whether the type of scanner used at baseline was associated with AVC presence or score. Neither presence of AVC at baseline (OR 0.95, 95% CI 0.64–1.41) nor baseline AVC score (β 0.63 AU, 95% CI −0.32; 1.23) were related to the type of scanner used.

## Discussion

Our study is the first to evaluate the relationship of Lp(a) with baseline presence, new onset, and progression of AVC in a population-based cohort during a median follow-up of 14 years. We demonstrate that Lp(a) is associated with baseline and new-onset AVC, but not with AVC progression. In individuals with AVC at baseline, the AVC score was the only determinant of further progression. These data imply that Lp(a)-lowering interventions may effectively prevent AVC onset but are less likely to attenuate AVC progression in individuals with established AVC (*Structured Graphical Abstract*).

### Lipoprotein(a) drives initiation, but not propagation of aortic valve disease

Genetic and epidemiological studies firmly support a causal role for Lp(a) in the pathophysiology of AVS. Lipoprotein(a) elicits osteogenic transformation of valvular interstitial cells predominantly via its oxidized phospholipid (OxPL) load, which can be counteracted by the addition of E06 antibodies blocking the OxPL epitope.^18,19^ In support, we observed that Lp(a) is robustly associated with baseline and new-onset AVC, independent of traditional risk factors. In contrast, even though most individuals in this population-based study had only minor AVC undetectable by routine echocardiography, we could not establish an impact of Lp(a) on AVC progression. It is known that increasing valvular calcium burden accelerates disease progression,^20^ eventually overruling the impact of initiating risk factors in severe AVS, but we are the first to show that even minor AVC progresses independently of Lp(a). These findings have major implications for the design of future trials, which have traditionally selected patients with mild-to-moderate AVS. Aortic valve calcification scores of such patients are typically over 10-fold higher than in the present study, making it plausible that progression is even more strongly determined by valvular calcium burden than by traditional risk factors.

### Progression of aortic valve calcification

Two previous longitudinal echocardiography and positron emission tomography studies have suggested Lp(a) may accelerate AVS progression.^18,21^ There are several distinct differences with the present study that deserve consideration. Both previous studies investigated the effect of Lp(a) in individuals with established AVS, whereas the current study setting is the apparently healthy general population. This may have led to differences in the type of aortic valve disease. In our AVC progression analysis, every individual had AVC at baseline, while the previous studies may also have included patients with valvular fibrosis, next to those with calcification. Furthermore, the primary outcome in the previous analyses were haemodynamic progression on echocardiography and a combined endpoint consisting of AVR and cardiac death vs. AVC progression on CT in the current study. Haemodynamic progression may have partially been driven by further valvular fibrosis, whereas the higher occurrence of cardiac death may be contributed to the relationship of Lp(a) with atherosclerotic cardiovascular disease. Finally, there is a large difference in follow-up duration: 1.5–2 years in the previous studies compared with 14 years in our study. If Lp(a) would truly drive progression, one would have expected this longer follow-up duration to also lead to acceleration of valvular calcium deposition, rather than only haemodynamic progression. To date, there is only one large population-based study (MESA) which evaluated AVC progression after a median follow-up of 2.4 years.^12^ This study showed that traditional cardiovascular risk factors are associated with AVC onset, but in patients with AVC at baseline, all these factors lose significance after adjustment for baseline AVC score. Likewise, Lp(a) was robustly associated with AVC onset in the present study, more so than traditional risk factors, but we could not establish an effect of Lp(a) on AVC progression. This observation supports the concept of two distinct disease stages in the pathophysiology of AVS: an initiation phase triggered by traditional cardiovascular risk factors, including Lp(a), and a propagation phase, during which calcium deposits in the valve inflict further damage, accelerating AVS progression largely independent of initiating risk factors.

### Future perspectives

What is the consequence of the absence of an association between Lp(a) and AVC progression? We have learned from previous randomized trials that LDL-C lowering in patients with mild-to-moderate AVS did not attenuate progression.^13–15^ In line, we found no association between non-HDL-C and hypertension and AVC progression in the present study. Although we found a more pronounced effect of Lp(a) than traditional risk factors on baseline and new-onset AVC, we observed no relationship with AVC progression after adjusting for baseline AVC score. Thus, in patients with established AVC, even potent Lp(a)-lowering strategies may not attenuate disease progression, which requires a shift of focus to the pre-calcific stages of aortic valve disease. However, this brings a critical logistical challenge, as a highly different approach for future randomized trials would be required. Instead of Lp(a) lowering in individuals with AVC, trials could select individuals at a high risk of developing AVC. We have previously shown that approximately one in five individuals with Lp(a) >50 mg/dL develop AVC between 50 and 60 years.^22^ The recommendation by the 2019 ESC/EAS dyslipidaemia guidelines to measure Lp(a) at least once in each person’s lifetime would facilitate selection of these high-risk individuals.^23^ Delaying the onset of AVC may be able to prevent end-stage AVS from occurring in these individuals, supported by our observation that not a single participant without AVC on the first scan underwent AVR during 14 years of follow-up.

### Study limitations

There are several limitations of the present study that deserve consideration. First, only a third of individuals who received the first CT scan underwent follow-up imaging, which may have resulted in a bias towards a healthier subpopulation of the original study cohort, potentially obscuring an effect of Lp(a) on AVC progression. Second, different CT scanners were used, but sensitivity analyses showed that the type of scanner was not related to presence nor progression of AVC. Moreover, previous studies demonstrated excellent inter-scanner variability between MDCT and DSCT.^24^ Third, we did not perform echocardiography of the aortic valve; therefore, we did not have data on haemodynamic progression, aortic valve orifice, or aberrant valvular morphology. However, in this general population cohort, the majority of individuals would have no detectable aortic valve disease on echocardiography, rendering CT the most sensitive imaging modality in the current study. Furthermore, as fewer than 1% of the population has aberrant valve morphology,^25^ we do not think that this significantly impacted our results. Fourth, sample storage at −80°C for up to 16 years may have led to reductions in Lp(a), particularly in high Lp(a) individuals.^26^ The majority of previous large population studies were hampered by this same limitation, but found robust associations between Lp(a) and the presence of AVC or AVS. Likewise, we found a robust association between baseline and new-onset AVC, making it less likely that Lp(a) degradation is responsible for not finding a relationship between Lp(a) and AVC progression. Fifth, triglyceride levels were not measured, which made us unable to calculate LDL-C levels. Accordingly, we adjusted for non-HDL-C. Finally, imaging was only performed at two time points, 14 years apart; it may be that Lp(a) accelerates disease progression in the early phases of calcification, but after years of progression, the valvular calcium burden overrides this effect.

## Conclusion

We demonstrate that Lp(a) is associated with baseline and new-onset AVC but not AVC progression in a population-based longitudinal imaging study with over 14 years of follow-up. These data challenge the concept that Lp(a) drives the progression of existing valvular calcification and warrant investigation of Lp(a)-lowering treatment in the pre-calcific stages of aortic valve disease.

## Supplementary material

Supplementary material is available at *European Heart Journal* online.

## Supplementary Material

ehac377_Supplementary_Data

## Data Availability

The data underlying this article will be shared on reasonable request to the corresponding author.
