# Supplementary material for: Lipoprotein(a) is associated with the onset but not the progression of aortic valve calcification
Source: Eur Heart J. 2022 Jul 23;43(39):3960–7. doi: 10.1093/eurheartj/ehac377 (PMC9840475; doi:10.1093/eurheartj/ehac377)
Supplement: ehac377_Supplementary_Data [file ehac377_supplementary_data.docx]

**Supplemental Table 1. Baseline characteristics – total study cohort vs current analysis**

|  | Total study cohort (n=5,129) | Participants included in the current analysis (n=922) | p value |
| --- | --- | --- | --- |
| Age (years) | 73.4±8.0 | 66.0±4.2 | <0.001 |
| Male | 2961 (57.7) | 440 (47.7) | <0.001 |
| Active smoker | 764 (15.2) | 114 (12.7) | 0.049 |
| Body mass index (kg/m^2^) | 27.6 (4.1) | 27.6 (3.7) | 1.000 |
| Systolic blood pressure (mmHg) | 149±21 | 143±18 | <0.001 |
| Diastolic blood pressure (mmHg) | 80±11 | 81±10 | 0.010 |
| Total cholesterol (mmol/L) | 5.63±1.00 | 5.83±0.96 | <0.001 |
| High-density-lipoprotein cholesterol (mmol/L) | 1.45±0.40 | 1.46±0.38 | 0.481 |
| Non-high-density-lipoprotein cholesterol (mmol/L) | 4.17±0.98 | 4.36±0.95 | <0.001 |
| Lipoprotein(a) (mg/dL) | 12 [6, 37] | 12 [5, 37] | 0.904 |
| Creatinine (mmol/L) | 77±16 | 80±15 | <0.001 |
| Use of blood pressure lowering medication | 2331 (45.9) | 294 (32.3) | <0.001 |
| Use of lipid lowering medication | 1154 (22.7) | 194 (21.3) | 0.349 |

Values are original, non-imputed data, depicted as mean±standard deviation for normally distributed data, median [interquartile range] for non-normally distributed data, and as number (percentage) for categorical data.
